# Effect of music and yoga on stress among Indian women during pregnancy

**DOI:** 10.6026/973206300211454

**Published:** 2025-06-30

**Authors:** Rohit Divya Gunvanthbhai, Siva Subramanian N., Mahalakshmi B.

**Affiliations:** 1 Depaertment of obstetrics and Gynaecological Nursing, Sankalchand Patel university, Visnagar, Gujarat - 384315, India; 2 Department of Psychiatric Nursing, Nootan College of Nursing, Sankalchand Patel University, Visnagar, Gujarat - 384315, India; 3 Department of Paediatric Nursing, Nootan College of Nursing, Sankalchand Patel University, Visnagar, Gujarat - 384315, India

**Keywords:** Antenatal women, stress during pregnancy, yoga therapy, music therapy, complementary therapies, perceived stress scale, non-pharmacological intervention

## Abstract

Effect of yoga and music on stress among antenatal Indian women Gujarat is of interest. Hence, a quasi-experimental design was used among 180 women divided equally into Yoga and Music groups. Interventions were delivered over two weeks (Yoga) and one week (Music), with stress measured using the Perceived Stress Scale. Both therapies significantly reduced stress. However, yoga showed a greater improvement (mean difference: 1.889 vs. 1.111). Correlation analysis revealed a stronger relationship in the music group, though overall effect was higher in the yoga group. These findings support integrating complementary therapies into prenatal care.

## Background:

Pregnancy is a profound phase in a woman's life, involving significant physiological and psychological transformations. Stress during pregnancy is a common experience and can stem from hormonal changes, lifestyle adjustments, and the anticipation of childbirth. If unmanaged, elevated stress levels can negatively impact both maternal and fetal outcomes, potentially leading complications such as preterm labor, low birth weight, and developmental delays [1]. Complementary therapies such as Yoga Therapy and Music Therapy have gained growing attention as safe, non-pharmacological strategies to reduce stress during pregnancy. Yoga, a holistic mind-body practice incorporating physical postures (asanas), breathing techniques (pranayama), and meditation, has been shown to alleviate stress and enhance physiological well-being among pregnant women [2]. Research demonstrates that prenatal yoga significantly lowers perceived stress levels and improves mood and physical health outcomes [3]. Additionally, immediate physiological benefits from yoga interventions, including reductions in salivary cortisol and alpha-amylase concentrations, have been observed [4]. Yoga's ability to regulate the hypothalamic-pituitary-adrenal (HPA) axis is believed to play a key role in mitigating stress responses [5]. Music Therapy, another accessible complementary intervention, involves the therapeutic use of music to induce relaxation, reduce anxiety, and uplift mood. Listening to calming music during pregnancy has been shown to decrease psychological distress and stress biomarkers [6].

Clinical studies suggest that even short-term music therapy interventions can significantly lower stress scores among pregnant women [7]. Interestingly, integrating yoga and music interventions may offer synergistic benefits. A study by Muzik *et al.* [8] reported that combining yoga with music significantly improved emotional well-being, maternal bonding, and reduced depressive symptoms more than either intervention alone. Yoga and music therapies are believed to exert their stress-reducing effects by modulating the autonomic nervous system and decreasing activation of the HPA axis, leading to improved parasympathetic activity and emotional stability [5]. Therefore, it is of interest to assess the effect of music and yoga on stress among Indian women during pregnancy.

## Methodology:

## Research design:

This quasi-experimental study employed a quantitative approach to evaluate the effectiveness of complementary therapies on stress during pregnancy. Two intervention groups were compared: one received Yoga Therapy and the other Music Therapy.

## Study setting and sampling:

The study was conducted in selected urban areas of Gujarat among antenatal women attending prenatal clinics. A total of 180 antenatal women were recruited using a convenience sampling method, with 90 participants in each group.

## Inclusion and Exclusion criteria:

Inclusion criteria were antenatal women between 20-34 weeks of gestation with low to moderate perceived stress and mild to moderate depression levels. Women with high-risk pregnancies or severe psychiatric disorders were excluded from the study.

## Intervention details:

Participants assigned to the Yoga Therapy group underwent a structured prenatal Yoga program, comprising gentle asanas, breathing exercises, and loosening techniques. Each session lasted 30 minutes and was conducted daily over a two-week period under professional supervision. Conversely, participants in the Music Therapy group listened to calming instrumental music for 30 minutes daily over one week, in a relaxed setting.

## Data collection tools and procedure:

Data were collected using a structured demographic questionnaire and the standardized Perceived Stress Scale (PSS). Pre-test assessments were conducted before the intervention, and post-test assessments were carried out after two weeks for the Yoga group and after one week for the Music group.

## Statistical analysis:

Descriptive statistics were used to summarize demographic characteristics. Paired sample t-tests were performed to compare pre-test and post-test scores within each group, and independent t-tests were used for between-group comparisons. A significance level of p < 0.05 was considered statistically significant.

## Results:

Table 1 (see PDF) show that most participants were aged 22-25, from middle-class families, and unemployed. The Yoga group had more postgraduates and nuclear families, while the Music group had more joint families. Primigravida women were the majority in both groups, with very few reporting previous abortions. Table 2 (see PDF) shows that the mean stress score in the Yoga Therapy group decreased from 14.22 to 12.33 with a mean difference of 1.889 (t = 3.622, p < 0.001), indicating a highly significant reduction. In the Music Therapy group, the mean stress score decreased from 14.00 to 13.11 with a mean difference of 1.111 (t = 1.993, p = 0.049), also showing a statistically significant reduction. Table 3 (see PDF) shows a positive moderate correlation (r = 0.433, p < 0.001) between complementary therapy and stress reduction in the Yoga group, and a positive strong correlation (r = 0.789, p < 0.001) in the Music group. Figure 1 (see PDF): The graph shows the distribution of antenatal women with low, moderate, and high stress levels before and after Yoga Therapy and Music Therapy interventions. Both groups demonstrated a reduction in moderate stress and an increase in low stress post-intervention, with greater improvement observed in the Yoga group. Figure 1 (see PDF): The graph shows the distribution of antenatal women with low, moderate, and high stress levels before and after Yoga Therapy and Music Therapy interventions. Both groups demonstrated a reduction in moderate stress and an increase in low stress post-intervention, with greater improvement observed in the Yoga group.

## Discussion:

The present study evaluated the effectiveness of Yoga Therapy and Music Therapy on stress reduction among antenatal women. Both interventions resulted in significant stress reduction, with Yoga Therapy demonstrating a greater impact compared to Music Therapy. These findings support the role of non-pharmacological therapies in promoting psychological well-being during pregnancy. Stress is a common phenomenon in pregnancy, linked to hormonal, emotional, and social changes. Elevated stress levels are associated with adverse outcomes such as preterm birth and low birth weight [9]. Safe and effective stress-reduction strategies are therefore essential. Our study demonstrated an increase in low stress levels post-intervention, especially in the Yoga group, consistent with findings by Araújo and Prado [10] and Narendran *et al.* [11], who reported significant reductions in stress among women practicing yoga. Biological studies reinforce these findings. Kusaka *et al.* observed reductions in salivary cortisol levels after prenatal yoga [12], while Mooventhan reviewed evidence that yoga improves parasympathetic activity and mental health [13]. Kiselev also confirmed reduced anxiety levels among pregnant women practicing yoga [14]. Comparing the two therapies, Yoga showed a greater mean difference in stress reduction (1.889) compared to Music Therapy (1.111). Previous studies confirm yoga's superior effectiveness. Roy *et al.* emphasized yoga's comprehensive benefits in prenatal mental health [15], while Field reported that yoga interventions help regulate the hypothalamic-pituitary-adrenal (HPA) axis, thus reducing stress responses [16]. Corrigan *et al.* confirmed through a meta-analysis that yoga reduces prenatal stress, anxiety, and depression [17], while Satyapriya *et al.* demonstrated improvements in heart rate variability and decreased stress among pregnant women following yoga practice [18^18^]. Music Therapy also significantly reduced stress, although the reduction was smaller. Studies by Chang *et al.* and Shafqat *et al.* indicated that music interventions effectively decrease psychological distress and anxiety in pregnant women [19, 20]. Furthermore, Shamkuwar and Asokan found that yoga outperformed music therapy in reducing antenatal stress [21].

Regarding correlation findings, a positive moderate correlation (r = 0.433) was noted for Yoga and a strong positive correlation (r = 0.789) for Music Therapy. This suggests that while Music Therapy had a strong relationship with stress reduction, Yoga Therapy produced greater overall improvement. Similar findings were noted by Bapat *et al.*, who demonstrated reductions in anxiety and perceived stress after yoga sessions [22]. Beddoe and Lee also found that mind-body interventions during pregnancy, including yoga, significantly reduced maternal stress and improved emotional resilience [23]. Overall, the findings affirm that Yoga Therapy offers holistic advantages, reducing stress and enhancing both physical and emotional resilience. Incorporating yoga into routine antenatal care programs can significantly improve maternal and fetal outcomes. This recommendation aligns with systematic reviews by Corrigan *et al.* and Mooventhan, emphasizing yoga's role in promoting healthier pregnancies [17, 13]. Future research should focus on larger, randomized trials to confirm these findings and develop standardized yoga modules tailored for pregnant populations.

## Conclusion:

Data show that both music and yoga significantly reduced stress. However, yoga showed a greater improvement. Correlation analysis revealed a stronger relationship in the music group, though overall effect was higher in the yoga group. These findings support integrating complementary therapies into prenatal care.
